# Analysis of 50 Neurodegenerative Genes in Clinically Diagnosed Early-Onset Alzheimer’s Disease

**DOI:** 10.3390/ijms20061514

**Published:** 2019-03-26

**Authors:** Vo Van Giau, Vorapun Senanarong, Eva Bagyinszky, Seong Soo A. An, SangYun Kim

**Affiliations:** 1 Department of Bionano Technology, Gachon University, Sungnam 13120, Korea; giauvvo@gmail.com (V.V.G.); navigator120@gmail.com (E.B.); 2 Department of Medicine, Mahidol University, Bangkok 10700, Thailand; vorasenanarong@yahoo.com; 3 Department of Neurology, Seoul National University College of Medicine and Neurocognitive Behavior Center, Seoul National University Bundang Hospital, Sungnam 13620, Korea

**Keywords:** Alzheimer’s disease, EOAD, next generation sequencing, 50 genes, Thailand

## Abstract

Alzheimer’s disease (AD), Parkinson’s disease (PD), frontotemporal dementia (FTD), amyotrophic lateral sclerosis (ALS), Huntington’s disease (HD), and prion diseases have a certain degree of clinical, pathological, and molecular overlapping. Previous studies revealed that many causative mutations in AD, PD, and FTD/ALS genes could be found in clinical familial and sporadic AD. To further elucidate the missing heritability in early-onset Alzheimer’s disease (EOAD), we genetically characterized a Thai EOAD cohort by Next-Generation Sequencing (NGS) with a high depth of coverage, capturing variants in 50 previously recognized AD and other related disorders’ genes. A novel mutation, *APP* p.V604M, and the known causative variant, *PSEN1* p.E184G, were found in two of the familiar cases. Remarkably, among 61 missense variants were additionally discovered from 21 genes out of 50 genes, six potential mutations including *MAPT* P513A, *LRRK2* p.R1628P, *TREM2* p.L211P, and *CSF1R* (p.P54Q and pL536V) may be considered to be probably/possibly pathogenic and risk factors for other dementia leading to neuronal degeneration. All allele frequencies of the identified missense mutations were compared to 622 control individuals. Our study provides initial evidence that AD and other neurodegenerative diseases may represent shades of the same disease spectrum, and consideration should be given to offer exactly embracing genetic testing to patients diagnosed with EOAD. Our results need to be further confirmed with a larger cohort from this area.

## 1. Introduction

Dementia is a clinical state caused by neurodegeneration and is characterized by a loss of function in cognitive domains and undesirable changes in behavior. Several types of neurodegenerative diseases were described [1,2,3,4], including Alzheimer’s disease (AD), Parkinson’s disease (PD), frontotemporal dementia (FTD), amyotrophic lateral sclerosis (ALS), prion diseases, and Huntington’s disease, which may share clinical and pathologic features [5]. The common feature of the neurodegenerative diseases could be the abnormal protein aggregates in the central nervous system [6]. Clinical and pathological similarities were observed in these diseases [1], suggesting that different types of dementia may be caused by overlapping genetic factors [6,7]. In addition, since these diseases have complex genetic backgrounds, genetic profiling is essential for an exact diagnosis and for the estimation of the future risk [1,4].

AD is the most common age-related neurodegenerative disorder in old adults (50% to 75%), affecting 23 to 35 million individuals worldwide [4,8,9,10]. Age is the most prominent biological risk factor and is used to classify AD patients into early-onset (EOAD, ≤ 65 years) and late-onset (LOAD, > 65 years) groups. Three genes are well known to be involved in the pathogenesis and onset of EOAD—the amyloid precursor protein (*APP*) (MIM #104760) [11,12], presenilin 1 (*PSEN1*) (MIM #104311) [13,14,15], and presenilin 2 (*PSEN2*) (MIM #600759) [16]. However, rare high-penetrant mutations in *APP*, *PSEN1*, and *PSEN2* explain only a small percentage (5–10%) of EOAD cases [1,17], leaving a large group of resolved pedigrees and a large number of genetically unexplained patients with EOAD, indicating that additional causal genes remain to be identified [18]. Meanwhile, Genome-Wide Association Studies (GWAS) identified or confirmed > 20 *loci* associated with LOAD, which were grouped in three major biological pathways—lipid metabolism, immune system, and synaptic dysfunction/cell membrane processes (e.g., endocytosis) [1,19,20].

On the other hand, the overlapping clinical and neuropathologic features of AD and other neurodegenerative forms of dementia lead to a misdiagnosis in 17–30% of AD cases [21]. In addition, despite the discovery of many genes responsible for AD, a large proportion of the genetic component of this disorder remains unexplained [22]. Hence, for each new genetic discovery in AD, patients and their family members may ask questions about whether genetic testing for “the new AD gene” is available. This raises the question of whether genetic risk factors other other neurodegenerative diseases may play a role in EOAD. Hence, a genetic overlap among all these neurodegenerative disorders cannot be ignored and may certainly be underestimated since most of the previous studies focused only on three genes (*APP*, *PSEN1*, and *PSEN2*) in EOAD cases, not covering the full spectrum of genes and leading to the missing heritability in the disease. To fill this gap in our knowledge, a gene panel with 50 genes involved in familial forms of neurodegenerative disorders was developed in prior research [1,4]. Ion Torrent Personal Genome Machine (PGM) system was launched in 2011 by Life Technologies and has been demonstrated as a more rapid, more sensitive, and less costly system, allowing higher throughput sequencing [4,14]. We performed Next-Generation Sequencing (NGS) on eight Thai patients with EOAD, with or without family history of disease and between 41 to 60 years of age, using the previous targeted exome panel of 50 genes.

## 2. Results

**Figure 1:**
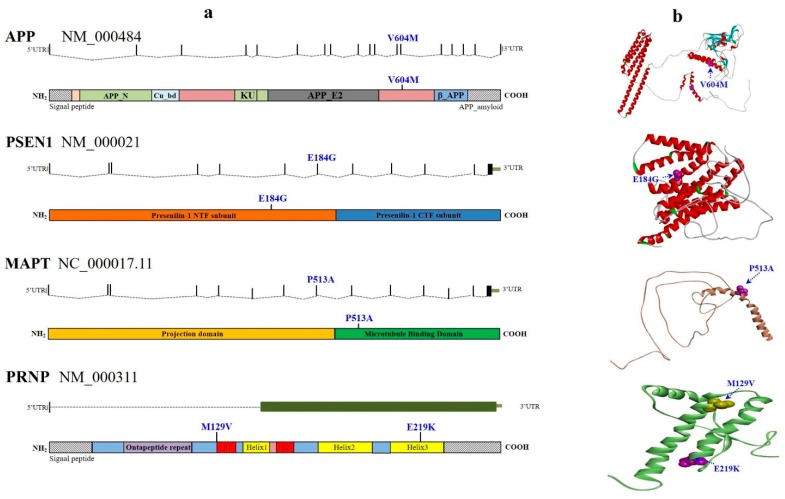
Schematic representation of the missense mutations in definite early-onset Alzheimer’s disease (EOAD) cases. Panel (**a**) shows the position of causative selected-mutations and coding risk variants relative to the respective gene and protein sequences. Panel (**b**) illustrates the position of each mutated amino acid residue relative to the 3D protein or domain structure. APP, Amyloid-beta precursor protein; Cu_bd, copper-binding domain; APP_E2, E2 domain of amyloid-beta precursor protein; KU, Kunitz-type serine protease inhibitor domain; β_APP, Beta-amyloid precursor protein; UTR, untranslated region; APP_N, N-terminal fragment of the β-amyloid precursor protein.

We identified 206 variants (non-synonymous, synonymous, intronic, and UTRs) and 18 indels (coding and intronic) in the genes studied. After variant filtering and verifying, a total of 63 missense mutations were discovered among 23 genes (*APP*, *PSEN1, CR1, TREM2, CTNNA3, DNMBP, SORL1, BACE1, LRP6, ABCA7, CD33, PINK1, PARK2, LRRK2, SIGMAR1, MAPT, ALS2, FIG4, OPTN, SPG11, CSF1R, NOTCH3*, and *PRNP*) out of the 50 analyzed genes in the eight Thai EOAD patients examined. The prevalence of genetic variants tended to be higher in cases with a strong family history than in sporadic ones (Table S1 and Figure 1).

### 2.1. Novel and Known AD Pathogenic Variants

Mutations in *APP, PSEN1*, and *PSEN2* genes are known to be autosomal dominant with complete penetrance in EOAD—however, we found that the variants discovered in this study did not always present complete penetrance or segregate perfectly with the disease status. First, the previously reported pathogenic *PSEN1* p.E184G mutation from two French families [23] was also detected in a Thai EOAD case at the disease onset. This known mutation was identified in a 41-year-old female (#7), who complained of her forgetfulness and slowness of thought. Her Mini-Mental State Examination (MMSE) was 29/30 and her Montreal Cognitive Assessment (MoCA) score was 25/30. Family history over more than four generations revealed dementia in several members, especially in her parents (age at onset (AAO), 59 years). This shows that the mutation was associated with a strong family history of dementia. No additional pathogenic mutations were found in other dominant causative genes of AD, FTD, or of other neurodegenerative diseases, such as *APP*, *PSEN2*, *PRNP*, *PGRN*, or *MAPT* (Table S1). Noticeably, after further examination of the clinical history of this patient with EOAD, we detected a reported family history of dementia. This pathogenic mutation, *PSEN1* p.E184G, was not found in the KRGDB, ExAC, and 1000Genomes databases, suggesting that it is the first mutation in Asian populations. In addition, despite *PSEN1* p.E184G being a known pathogenic mutation involved in early-onset AD, it is also involved in the frontal variant of EOAD. Amino acid at position 184 is likely an important residue in PSEN1 since both p.E184G and p.E184D may be involved in the onset of EOAD.

Since mutations in *APP* were limited, only few variants were studied in detail. A possible novel variant, p.V604M, was identified in exon 14 of the *APP* gene, which was predicted to be pathogenic by all these in silico predictions [24]. Subject #6 developed progressive non-fluent aphasia in 2011 and DDx Alzheimer’s disease (logopenic aphasia) from the age of 55 years, accompanied by a slight increase in the degree of right hippocampal atrophy and an increase in diffuse cortical brain atrophy to a moderate degree. Family history revealed dementia only in the subject’s father, family history for the rest of the members over more than three generations was negative for neurological diseases, suggesting this is a sporadic case. In addition, the level of three common proteins associated with AD patients—Aβ42, tTau, and pTau—in cerebrospinal fluid (CSF) were further analyzed, their levels were 555 pg/mL, 285 pg/mL, and 37 pg/mL, respectively. No available database has reported this mutation. No additional pathogenic mutations were identified in other dominant causative genes of AD, FTD, or of other neurodegenerative diseases, such as *PSEN1, PSEN2, PGRN*, or *MAPT* on this patient.

### 2.2. Known FTD Pathogenic Variant

Mutations in FTD genes are also known to segregate in a dominant pattern. Among the seven FTD genes analyzed, we observed only one pathogenic variant in the microtubule-associated protein tau gene (*MAPT*, c.1537 C>G, p.P513A), which was discovered in a male patient (#2) who developed progressive slowing in memory with an onset age of 51 years, while no pathogenic mutation was found in *APP, PSEN1*, or *PSEN2* genes. Clinically, the *MAPT* (c.1537 C>G, p.P513A) was a rare variant mutation. The patient (#2) struggled with difficulties in daily activities, presented impairment in speech and comprehension, and was diagnosed with AD and logopenic aphasia. He received treatment with Rivastigmine (12 mg/day) and Memantine (20 mg/day). Magnetic resonance imaging (MRI) showed mild cerebral atrophy with multiple lacunar infractions. Brain single photon emission computed tomography (SPECT) revealed decreased cerebral perfusion at the bilateral temporal, parietal, and bilateral anterior frontal areas. His APOE genotype was ε3/ε3. *MAPT* p.P513A was first reported in a Chinese family with progressive non-fluent aphasia [24]. The proband was a 52-year-old male, whose main symptoms were difficulty in naming and finding the correct words. He also showed slight memory impairment but no major cognitive decline (MMSE = 29/30). Disease was segregated with the mutation since P513A was only present in the family members presenting similar phenotypes. This mutation was predicted as damaging by PolyPhen-2, with a 0.823 HumDiv score. *MAPT* p.P513A may be a rare variant in South Asian populations, including Thai populations, because it was reported to be present in low frequency in South Asia (1/8214 allele count, 0.0001217 allele frequency) in the Exome Aggregation Consortium (ExAC, http://exac.broadinstitute.org/).

### 2.3. Known PD Pathogenic Variants

Mutations in PD-associated genes present different patterns of segregation. *PARK2*, *PARK7*, and *PINK1* are known to cause early-onset PD with a recessive inheritance mode, while *SNCA* and *LRRK2* are known to cause dominantly inherited PD [25]. We detected 12 different novel and known pathogenic PD variants in *PINK1* (p.Y253D, p.A340P, and p.N521T), *PARK2* (p.S167N and p.V380L), and *LRRK2* (p.R50H, p.N551K, p.R1398Q, p.S1647T, p.R1628P, p.N2081D, and p.M2397T) in eight different carriers, all of whom were heterozygous for the variant.

The three *PINK1* variants were also detected in patient #6, with EOAD (AAO = 55 years), whereas p.A340P and p.N521T were identified in patient #1 (AAO = 60 years). Interestingly, all three variants were missing in KRGDB, ExAC, and 1000Genomes databases, supporting the fact that they are novel variants. Among the mutations found, both p.S167N and p.V380L were detected in *PARK2*, which had been previously reported [26]. These already known mutations were found in all patients, with the exception of patient #2. Mutations in *PARK2* and *PINK1* are known to cause early onset PD (AAO = 12–58 years) with a recessive pattern of inheritance. All the individuals reported here had an AAO < 60 years, suggesting that these *PARK2* and *PINK1* variants can be causative of AD.

Since *LRRK2* mutations have been associated with an AD-like pathology, there may be a partial overlap between neurodegenerative pathways in both AD and PD [27,28,29]. Seven variants were detected in *LRRK2*, all of which could be common variants, with a putative PD risk factor (Table S1). Mutations p.R1628P and p.N551K were associated with PD among Chinese patients [30]. In addition, the p.N551K variant did not appear to modulate the risk of AD [31], whereas it was found in a 51-year-old patient (#4) with EOAD in the present study. Only one patient carried the *LRRK2* p.R1398Q mutation, which resulted in decreased kinase activity. Although mutations in the *LRRK2* gene are the most common genetic cause of PD, the penetrance of the variants observed here is surprising. These findings provide evidence that *LRRK2* exonic variants may co-contribute to susceptibility to AD. Further validation with large series and meta-analysis studies is of high importance.

### 2.4. Other Novel and Known Dementia Variants

Recent meta-analysis of GWAS identified at least 22 genes implicated in LOAD, as described above, that are potentially implicated in AD pathogenesis [32], though their functional role and significance still have to be fully elucidated. Given the negative for mutations in the three EOAD genes (*APP, PSEN1*, and *PSEN2*), we additional identified 30 non-synonymous variants in the LOAD-associated risk factor genes studied. Possible pathogenic mutations or risk variants were found in *CR1, TREM2, CTNNA3, DNMBP, SORL1, BACE1, LPR6, ABCA7*, and *CD33* (Table S1).

Interestingly, many studies have suggested that some mutations in *TREM2* (triggering receptor expressed on myeloid cells 2) correlate with a significantly increased risk of developing AD [33,34], leading to a substantial risk of developing AD in their mid-60s [35]. Here, the NGS study identified a heterozygous *TREM2* variant, p.L211P (rs2234256), that had recently been shown to increase AD risk. This mutation was discovered in patient #2 (AAO = 55 years), who also carried *MAPT* p.P513A, suggesting linkage disequilibrium. Thus, even in the presence of the rare variants in *TREM2* that may increase AD risk, TREM2-containing pathways play a significant role in the disease. *TREM2* p.L211P resides within the cytoplasmic domain, which can be led by the shorter TREM-2V transcript. A recent study demonstrated that *TREM2* p.L211P is strongly associated with an increased risk for AD in African Americans [36]. Although the functional consequences of the variant remain to be established, such findings pave the way to inquire alternative disease risk mechanisms and may, therefore, promote our understanding of the role of TREM2 in AD and other neurodegenerative diseases.

In addition, the overlapping of clinical and neuropathologic features between AD and other neurodegenerative forms of dementia may result in misdiagnosis in 17–30% of AD cases [1,4,21], only two rare (25%) high-penetrant mutations in *APP* and *PSEN1* were found in two EOAD patients, leaving 75% of the autosomal dominant pedigrees genetically unexplained in the cohort. To fill this gap in our knowledge, the NGS study also explored the frequencies and spectrum of mutations in genes previously implicated in neurodegenerative disorders. We could not classify the rest of the variants (found in the eight patients) in the dementia gene list as causative or not. For instance, two known mutations in *PRNP*, p.E219K and p.M129V, have been widely documented as risk factors for sporadic Creutzfeldt–Jakob disease (sCJD)—but both were associated with EOAD in this study, suggesting whether these common variants are benign polymorphisms or not [37]. Variants with unknown significance may be possibly reclassified in the future, based on putative functional and/or genetic arguments, and might confer an increased risk for developing AD.

On the other hand, the colony stimulating factor 1 receptor gene (*CSF1R*) is the only known gene that has been identified in hereditary diffuse leukoencephalopathy with spheroids (HDLS), which causes dementia, psychiatric symptoms, parkinsonism, seizures, and other neurological symptoms, and typically begins when patients are in their 40s and 50s [37]. Due to the overlapping of neurodegenerative pathologies between AD and other dementia-related diseases, this could lead to misdiagnosis. In this cohort, we found a novel P54Q mutation in exon 2 of the *CSF1R* gene. This variant was missing in KCDC, ExAC, and also in the 1000Genomes datasets. PolyPhen revealed this mutation as benign, with 0.145 HumDiv and 0.215 HunVar scores. Sorting Intolerant from Tolerant (SIFT) software suggested the mutation to be tolerated, with a score of 0.53. This mutation was not located on the pathogenic mutation cluster of the CSF1R protein (the tyrosine kinase domain (exon 13–21)), therefore, it is not known whether it may be involved in pathogenic mechanisms. Hence, *CSF1R* p.P54Q was not a causative factor for HDLS. More studies are needed to clarify whether *CSF1R* p.P54Q is important for an AD progression mechanism and if it is a genetic risk factor for AD.

A second probable novel variant, p.L536V, was identified in *CSF1R* at exon 11, located nearby the intracellular tyrosine kinase domain of CSF1R, which is encoded by exons 12–22. This mutation was also identified in a Thai patient with EOAD carrying *APP* p.V604M, as described above. *CSF1R* p.L536V is probably a novel mutation because it is missing in the KCDC database. However, it appeared in the ExAC database with the rare frequency of 0.00009984. PolyPhen2 analysis suggested that this mutation is probably a damaging variant, with 1.000 and 0.996 HumDiv and Hum-Var scores, respectively. Both Polyphen-2 and SIFT predictions strongly suggested L536V as a damaging variant.

Since these *CSF1R* mutations are located outside the intracellular tyrosine kinase domain, they may not to be risk factors for HDLS. However, we present the first report to date, of two cases associating *CSF1R* mutations with EOAD. Our data strongly support that both *CSF1R* p.P54Q and p.L536V present clinically with EOAD and that the patients have strong clinical characteristics. Additionally, further analyses on these mutations and their haplotype are required to further investigate whether the variants could be a hotspot for mutations in *CSF1R* associated with the clinical phenotype.

### 2.5. In Silico Gene/Protein Functional Interaction Network

**Figure 2:**
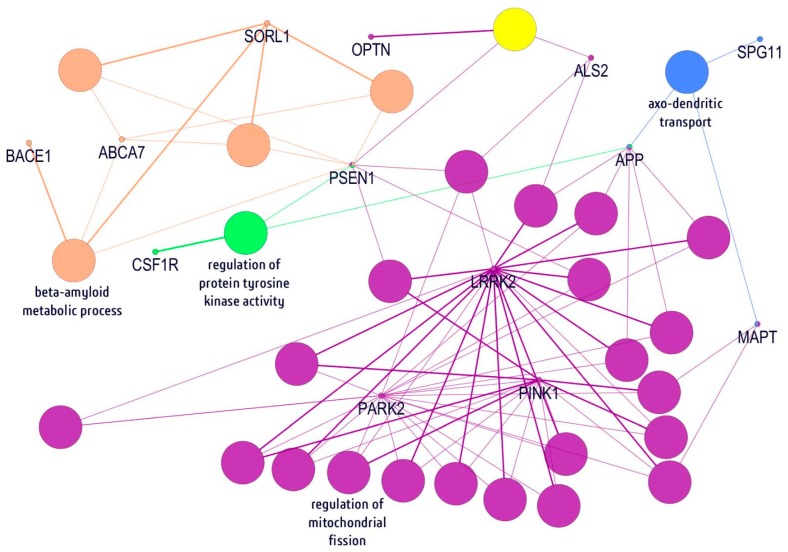
Visualization of neurodegenerative disorders of 23 genes in network modules. Each biological process was represented with the nodes which are connected with lines to indicate interactions. The orange nodes indicated beta-amyloid metabolic process genes, while regulation of protein tyrosine kinase activity and mitochondrial fission genes are presented as green/yellow and purple, respectively. The light-blue nodes revealed genes associated with axo-dendritic transport.

In order to understand the molecular basis of how different mutations within these genes can contribute to disease, an interaction network was performed using the ClueGo Cytoscape (Figure 2). ClueGo mapping suggested that the analyzed genes could be involved in multiple pathways, being associated with amyloid processing and metabolism (*PSEN1*, *SORL1*, *ABCA7*, and *BACE1*), regulation of tyrosine kinase activity (*OPTN1*), receptor recycling (*SPG11* and *MAPT*), and synaptic transmission or regulation of oxidative stress (*LRRK2*, *PARK2*, and *PINK1*). It also revealed that neurodegenerative disease-related pathways can be quite complex, and impairment of those metabolic functions may result in neurodegeneration.

## 3. Discussion

Recent reports reveal that rare mutations in *APP*, *PSEN1*, and *PSEN2*, cause, contribute, and modify the risk for AD [38,39]. Most cases of AD are suggested to be associated with complex mechanisms, including rare coding variants, epigenetics, or regulatory variants accounting for “missing heritability” [21]. Genetic mutations in AD and other common neurodegenerative diseases may overlap, suggesting a contribution toward AD risk by genes involved in other diseases, which has been sought for some time now. This is the first study that thoroughly examines pathogenic mutations in known neurodegenerative genes and evaluates the contribution of rare and common variants in these genes toward AD in a cohort of patients affected by sporadic and familiar EOAD. In addition, this study tested our hypothesis—through examination of our 50-gene panel—that there is a significant phenotypic overlap between sporadic/familiar EOAD and other neurodegenerative forms of dementia, which may be explained by a common genetic background [4]. Hence, to further characterize the genetic architecture of EOAD, NGS was employed and investigated the presence of rare coding variants in known neurodegenerative disorders genes from eight Thai patients with EOAD.

Although the study found many variants in the panel of 50 genes from the patients with EOAD, we only focused on known and well-characterized pathogenic mutations. Those variants were predicted as pathogenic, because they were initially found in EOAD cases and were not identified in the general population and/or by functional analyses. For instance, *PSEN1* p.E184G is a known pathogenic mutation involved in EOAD and also in the frontal variant of EOAD. Probably, amino acid 184 may be an important residue in PSEN1, because both E184G and E184D are involved in the onset of EOAD. Furthermore, although the patient carrying the PSEN1 p.E184G mutation was also homozygous for PRNP p.M129V, a common polymorphism and risk factor for sporadic Creutzfeldt–Jakob disease (sCJD) [40], p.M129V may be associated with AD in this patient. Interestingly, the PRNP129 variants (Met/Met, Met/Val, Val/Val) were suggested to be a possible disease modifier of AD [41]. Further studies should be carried out to assess the effects of PRNP129 in the AD phenotype in association with the *PSEN1* p.E184G mutation. It was unknown whether other variants or genes could be disease modifiers in the patients with EOAD since several variants in other risk factor genes in the panel were also discovered. In *APP*, no pathogenic mutation was found in exons 16–17, but a possible novel mutation, V604M, appeared in exon 14. This mutation was predicted as a possibly damaging variant, but emerging studies available on mutations show that it is located outside the amyloid-forming region [41,42]. Approximately 25 out of 32 coding mutations in *APP* were reported as pathogenic, and several of them are present in an autosomal dominant pattern in EOAD [43,44]. Interestingly, not only mutations located in critical regions of amyloid precursor proteins, including the region that generates Aβ, cause familial susceptibility to AD [11,45,46]. Several mutations outside the Aβ region also associated with familial Alzheimer’s, including the substituted amino acids 595 and 596, have been found to dramatically increase production of Aβ [46]. These results suggested that the V604M mutation may contribute to disease by a mechanism other than enhancing amyloid fibril formation, but further study is needed. On the other hand, potential risk mutations were found in patient #2, such as P513A in *MAPT*, which was suggested to play a role in progressive non-fluent aphasia, or *TREM2* p.P211L, which may be a risk modifier for AD/FTD. This mutation may have lower soluble TREM2 levels in CSF [47]. In addition, R1628P in *LRRK2* (patient #8) was previously shown as a PD risk mutation, but its role in AD should be further analyzed.

The clinical complexity of dementias has raised speculation that many other neurological diseases are misdiagnosed as AD [48]. Our study confirmed that screening patients for strong genetic factors involved in neurodegenerative diseases, such as AD, FTD, ALS, or PD is important [49]. There is a pathological overlap between neurodegenerative diseases in terms of clinicopathological features and brain lesions. It is possible that these diseases act through common disease mechanisms, or “neurodegenerative burden”, and mutations may increase the risk for neurodegenerative diseases [50,51]. Our data detected not only known pathogenic or risk mutations but also several variants whose significance is unclear. Further studies are required to analyze which variants can contribute to disease progression. In silico modeling and pathway analyses may help to understand how these genes work together and how they relate to each other in terms of disease progression (Figure 2). Our ClueGo pathway mapping suggested that the examined genes may function together through different pathways, such as amyloid metabolism, oxidative stress-associated mechanisms, or regulation of the axo-dendritic transport. On the other hand, although the study discovered many variants in the panel of 50 genes from patients with EOAD, due to the sample size we are still underpowered to detect a significant association for genes that we know are directly involved in the pathogenicity of the disease. However, the study has shown that, despite having in our cohort individuals carrying known and causal pathogenic variants, the current 50-gene-based analysis is useful for detecting these genes as being implicated in the disease. This is the first study involving a complex genetic screening among patients with EOAD in Thailand. Currently, only a few studies are available on EOAD in South-East Asia [12,14,16,52,53,54,55]. However, to improve disease diagnosis, genetic screening is important.

In summary, mutations in *APP*, *PSEN1*, and *PSEN2* genes are not always common in EOAD, therefore, genetic screening plays a pivotal role in differential diagnosis through other related disorder-associated genes and their pathway analysis. Genetically, many mutations from other neurodegenerative genes were found in patients with EOAD, leaving the definitive diagnosis a significant challenge, while all patients display clinical, neuroimaging, and neuropathological features meeting the diagnostic criteria for AD. Here, we report neuropathological confirmed patients with EOAD carrying likely pathogenic mutations that may be a potential association between AD and other related disorders. In addition, we support previous studies that suggest that the genetic architectures of AD, PD, and FTD/ALS can be overlapping [50,51,56]. Our study initially provides compelling evidence that AD and other neurodegenerative diseases may represent shades of the same disease spectrum. Given the very rare frequency of *APP*, *PSEN1*, and *PSEN2* pathogenic mutations detected in the screened patients (two individuals with EOAD), our hypothesis should encourage genetic screening in larger cohorts of both EOAD and LOAD, as well as dementia cases using the gene panel. Furthermore, our results suggest that genetic counseling and testing should be offered to patients diagnosed with EOAD [57], given the substantial proportion of pathogenic mutations and risk variant carriers discovered in this pathologically proven cohort.

Despite the limitations of this study, the analysis of its results was thorough and the results point in the expected direction. One of the limitations is that we focused on 50 disease-associated genes, while there are emerging publications on possible novel risk genes. In addition, we could not analyze copy number variants (e.g., *APP*), which could be important in disease progression. C9orf72 G4C2 promoter expansion was also not screened in the patients, however, it may be rare among Asians. An additional issue was the small sample size, as we analyzed only eight patients. However, the complex and heterogeneous presentation of AD makes it difficult to recruit large cohorts with good phenotypic characterization. Since all living family members and relatives refused genetic testing and declined to provide any additional information regarding their health, we were unable to perform segregation analysis on these mutations. In addition, we were unable to make in vivo or in vitro studies on the mutations found, which could confirm or refute the role of variants in disease [58]. Finally, these results provide further genetic evidence of the clinical, pathological, and molecular overlap between neurodegenerative diseases.

## 4. Materials and Methods

### 4.1. Study Samples

Eight patients (four females and four males) clinically diagnosed with EOAD were recruited from the Department for Neurodegenerative Diseases, Center for Neurology, Thailand. All patients were diagnosed with either definite or probable AD according to NINCDS-ADRDA (National Institute of Neurological and Communicative Disorders and Stroke and the Alzheimer’s disease and Related Disorders Association) and CERAD (Consortium to Establish a Registry for Alzheimer’s disease) guidelines [59]. The mean age of the subjects at onset was 55 years (range 41–60). An overview of the included subjects is shown in Table 1. All subjects were tested for APOE genotypes by the EzWay™ Direct PCR method (Komabiotech, Korea), comprising of the *APOE* ɛ2, ɛ3, and ɛ4 alleles. This study was conducted with approval from the Institutional Review Board of Seoul National University College of Medicine & Neurocognitive Behavior Center, Seoul National University Bundang Hospital, Korea (B-1302/192-006, approval date: 15/03/2013) and the Faculty of Medicine Siriraj Hospital, Mahidol University, Bangkok, Thailand. 

### 4.2. Selection of Candidate Genes, Variants, and Analysis

We focused our analysis on genes and variants reported as pathogenic and causing AD, FTD/ALS, PD, or prion diseases, as previously described [4]. For AD and FTD, we restricted our analysis to those genes listed in the AD and FTD mutation database (http://www.molgen.vib-ua.be/ADMutations/, accessed January 2019). For PD, we started off with those genes and variants listed in the PD mutation database (http://www.molgen.vib-ua.be/PDMutDB/, accessed January 2019). For other disorders, we restricted our analysis to those genes consistently reported in the literature as causative of familial ALS, HD, and prion diseases, since respective mutation databases are not available.

### 4.3. Ion Torrent PGM Sequencing and NGS Processing

**Figure 3:**
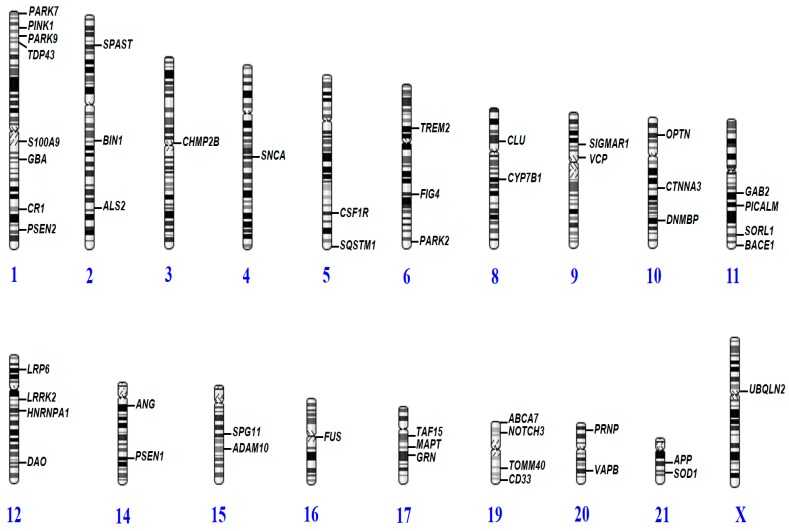
Distribution of neurodegenerative genes in the human genome where causative or probably causative variants were reported to cause early-onset dementia.

DNA was extracted from whole blood by standard laboratory methods. NGS approach by the Ion Torrent PGM system was performed by Theragen Etex Bio Institute (Seoul, Korea, http://www.theragenetex.com/), using a specifically designed gene panel of 50 causative and risk factor genes for various neurodegenerative disorders (Table 2 and Figure 3).

To confirm the presence of the identified mutations, standard sequencing was also performed in both directions using the same NGS primer sets [4]. Automated Sanger sequencing reactions were carried out by BioNeer Inc. (Daejeon, Korea). Big Dye Terminator Cyclic sequencing was performed using an ABI 3730XL DNA Analyzer (Bioneer Inc., Daejeon, Korea). Sequencing data were aligned using NCBI Blast (http://blast.ncbi.nlm.nih.gov/Blast.cgi), and chromatograms were screened by DNA BASER (http://www.dnabaser.com) software. The novelty of identified mutations was analyzed by screening for their inclusion in the Exome Aggregation Consortium (ExAC, http://exac.broadinstitute.org/) and 1000 Genomes (http://www.1000genomes.org/) databases. We also checked the mutations in the Korea Centers for Disease Control and Prevention (KCDC; http://www.cdc.go.kr), where DNA from 622 control individuals was sequenced by whole genome sequencing.

### 4.4. In Silico Gene/Protein Functional Interaction Network

Mutations were analyzed using PolyPhen-2 (http://genetics.bwh.harvard.edu/pph2/), SIFT (http://sift.jcvi.org/), and PROVEAN (http://provean.jcvi.org) algorithms software to predict the phenotypic impact of identified missense mutations. In addition, a 3D model was designed, comparing normal and mutant prion proteins (PrPs). Mutant and normal prion protein structures were constructed by the Raptor X web server (http://raptorx.uchicago.edu/), a protein structure prediction server using amino acid sequences. Discovery Studio 3.5 Visualizer, from Accelrys, was used to display the 3D images. Lastly, all the genes associated with discovered mutations and their ontology were further analyzed by ClueGO v2.0.5 to infer their functional influences in AD metabolic pathways. This tool allows the visualization of non-redundant biological networks of large clusters of genes grouped in functional networks with statistical evaluations—in respect to the existing annotations in the Gene Ontology [60].

## Figures and Tables

**Table 1:** Available demographic and clinical characteristics of 8 Thai patients with inherited early-onset Alzheimer’s disease (EOAD).

Subject No.#	Gender	Age (Years)	APOE	Family History	Clinical Diagnosis	Remark of the Imaging Findings
1	Male	60	ε3/ε3	No	AD	Brain single photon emission computed tomography (SPECT) showed decreased uptake at left frontal and left posterior temporal, posterior cingulate gyrus, and most severe at left parietal
2	Male	51	ε3/ε3	Yes	AD, logophenic aphasia	Magnetic resonance imaging (MRI) showed mild cerebral atrophy with multiple lacunar infraction. Brain SPECT revealed decreased cerebral perfusion at the bilateral temporal, parietal, and the bilateral anterior frontal areas
3	Male	56	ε3/ε3	Yes	AD	MRI brain showed diffused cerebral atrophy with minimal small vessel disease bilateral at hippocampal atrophy
4	Female	51	ε3/ε3	No	AD	MRI brain showed diffused cerebral atrophy with bilateral moderate hippocampal atrophy grade IIIEEG study was consistent with moderately severe diffused encephalopathy
5	Male	55	ε3/ε3	No	AD	MRI brain showed no specific white matter change at periventricular area, whereas SPECT revealed no cerebral perfusion abnormality
6	Female	55	ε3/ε3	Yes	AD, logophenic aphasia	MRI brain, moderate to severe atrophy of right hippocampusIMP, moderate to severe atrophy of right hippocampus
7	Female	41	ε3/ε3	Yes	Early-onset dementia	Mini-Mental State Examination (MMSE) was 29/30, Montreal Cognitive Assessment (MoCA) was 25/30
8	Female	60	ε3/ε4	No	Early-onset dementia with language impairment	MRI showed mild generalized cerebral cortical atrophy, with a small spot of nonrestrict diffusion. T2/FLAIR analysis revealed hypersignal intensity in the right subcortical frontal lobe

**Table 2:** List of 50 genes where causative or probably causative variants were reported to cause early-onset dementia.

Disease Categories	No. of Genes	Candidate Genes Selection
Alzheimer’s disease	19	*APP, PSEN1, PSEN2, S100A9, CR1, BIN1, TREM2, CLU, CTNNA3, DNMBP, SORL1, BACE1, PICALM, GAB2, LPR6, ADAM10, ABCA7, CD33, TOMM40.*
Amyotrophic Lateral Sclerosis (ALS)and Frontotemporal dementia (FTD)	18	*TDP43, CHMP2B, SIGMAR1, VCP, FUS, GRN, MAPT, UBQLN2, ALS2, TAF15, FIG4, OPTN, DAO, HNRNPA1, SOD1, ANG, VAPB, SQSTM1.*
Dementia with Lewy Bodies	7	*PINK1, PARK7, PARK9, GBA, SNCA, PARK2, LRRK2.*
Other neurodegenerative	6	*SPAST, CYP7B1, SPG11, CSF1R, NOTCH3, PRNP.*

## Supplementary Materials

Supplementary materials can be found at https://www.mdpi.com/1422-0067/20/6/1514/s1.
