# Supplementary material for: Analysis of 50 Neurodegenerative Genes in Clinically Diagnosed Early-Onset Alzheimer’s Disease
Source: Int J Mol Sci. 2019 Mar 26;20(6):1514. doi: 10.3390/ijms20061514 (PMC6471359; doi:10.3390/ijms20061514)
Supplement: Supplementary file 1 [file ijms-20-01514-s001.pdf]

Research article

Analysis of 50 neurodegenerative genes in clinically diagnosed early-onset Alzheimer's disease

Vo Van Giau[1a], Vorapun Senanarong [2a], Eva Bagyinszky[1], Seong Soo A. An[1+], SangYun Kim[3+]

[1]Department of Bionano Technology, Gachon University

[2]Department of Medicine at Mahidol University, Thailand

[3]Department of Neurology, Seoul National University College of Medicine & Neurocognitive Behavior Center, Seoul National University Bundang Hospital

[a]These authors contributed equally.

+ Address for co-correspondence: Seong Soo A. An, PhD and SangYun Kim, MD, PhD

Seong Soo A. An, PhD

Department of Bionano Technology, Gachon Bionano Research Institute, Gachon University, 1342 Sungnam-daero, Sujung-gu, Seongnam-si, Gyeonggi-do,

461-701, South Korea

Tel: +82-31-750-8755

Fax: +82-31-750-8755

E-mail: seong.an@gmail.com

SangYun Kim, MD, PhD

Department of Neurology, Seoul National University Bundang Hospital, Seoul National

University College of Medicine, 300 Gumidong, Bundang-gu, Seongnam-si, Gyeonggi-do,

463-707, South Korea

Tel:+82-31-787-7462

Fax: +82-31-719-6815

E-mail: neuroksy@snu.ac.kr

Table S1. ***Missense mutation implicated in EOAD identified by NGS genetic analysis of 08 cases***

(B: Benign; D: Damaging; T: Tolerant; N: Neutral; NA: Not Available)

**No.**	**Gene symbol**	**Protein change**	**SNP-ID**	**Frequency**				***In silico* prediction**			**Clinical interpretation AD mutation database**
				#ID Patient	***622 normal control***	***ExAC***	***1000 Genomes***	***PolyPhen2 HumDiv***	***Sift score***	***Provean***	
1	*APP*	p.V604M	NA	6	NA	NA	NA	0.474 (D)	0.095 (T)	-0.72 (N)	*May be involved in disease phenotypes*
2	*PSEN1*	p.E184G	NA	7	NA	NA	NA	0.73 (D)	0.005 (D)	-8.99 (D)	*Known AD-causative mutation*
3	*CR1*	p.H1658R	rs2274567	2, 3, 4 & 7	0.204180	0.2510	0.234	0.98 (D)	0.89 (T)	-1.81 (N)	*Possible risk factor for AD*
		p.T1858M	rs3737002	1, 2, 4, 8	0.425241	0.2750	0.069	0.995 (D)	0.019 (D)	-0.83 (N)	*Common variant, possible AD risk factor*
		p.T2060S	rs4844609	8/8	1	0.9853	0.99	0.95 (D)	0.019 (D)	0.55 (N)	*Common variant, possible AD risk factor*
		p.I2065V	rs6691117	2, 4, 5, 7	0.2202	0.3341	0.354	0.005 (B)	1 (T)	0.49 (N)	*Common variant, possible AD risk factor*
		p.P2277R	rs3811381	2, 4, 5	0.191318	0.2403	0.145	0.032 (B)	0.44 (T)	-0.61 (N)	*Unknown significance*
		p.T2419A	rs2296160	1,2,4,5,8	0.664791	0.8159	0.8431	0.001 (B)	0.98 (T)	-0.10 (N)	*Common variant*
4	*TREM2*	p.L211P	rs2234256	2	NA	0.01	0.03	0 (B)	0.131 (T)	-1.145 (N)	*Common variant, possible AD risk factor*
5	*CTNNA3*	p.H150R	NA	5	NA	NA	NA	0.93 (D)	0.016 (T)	-2.145 (D)	*May be AD risk factor*

		p.S596N	rs4548513	8/8	0.431672	0.4120	0.5120	0.001 (B)	1 (T)	2.04 (N)	*Possible risk factor for LOAD and autism*
6	*DNMBP*	p.N373K	rs35924554	1	0.01	0.09	0.05	0.2 (B)	0.721 (T)	1.04 (N)	*Unknown significance*
		p.R1137 Q	rs11629667 6	5	0.0040	0.0006	0.00005	0.99 (D)	0.006 (T)	-2.58 (D)	*Unknown significance*
		p.C1413W	rs11190305	1, 2, 4	0.261254	0.3442	0.288	0.094 (B)	0.18 (T)	-1.61 (N)	*Common variant*
		p.P1424 L	rs11357665 1	6	NA	0.0004	0.0002	0.0 (B)	0.721 (T)	1.61 (N)	*Unknown significance*
7	*SORL1*	p.A528T	rs2298813	1,3,4	0.17283	0.0721	0.08	0.59 (B)	0.3 (T)	0.12 (N)	*AD risk factor*
		p.Q1074 E	rs1699107	8/8	0.46	0.854	0.98	0.39 (B)	0.45 (T)	1.12 (N)	*Common variant, unknown significance*
		p.V1967 I	rs1792120	8/8	0.998392	0.9953	0.98	0.003 (B)	1 (T)	0.12 (N)	*Common, unknown*
8	*BACE1*	p.C481R	rs74642146	8/8	1.0	0.99	0.9	0 (B)	0.4 (T)	0.42 (N)	*Common mutation*
9	*LRP6*	p.V1062I	rs2302685	8/8	0.922830	0.8474	0.874	0 (B)	1 (T)	0.96 (N)	*Possible AD risk factor*
10	*ABCA7*	p.E188G	rs3764645	1,2,4,5,6,8	0.428457	0.4838	0.415	0.06 (B)	0.67 (T)	-0.87 (N)	*Unknown*
		p.G398D	NA	6	NA	NA	NA	0.8 (D)	0.25 (T)	-2.89 (D)	*unknown*
		p.R463H	rs3752233	7	0.163183	0.04775	0.05	0.8 (D)	0.25 (T)	-2.89 (D)	*unknown*
		p.N718T	rs3752239	3, 5, 7	0.1543	0.070	0.05	0.58	0.24	-5.17	*unknown*

								(D)	(T)	(D)	
		p.D964E	rs117390715	3	0.0080	0.001506	0.0027	1 (D)	0 (D)	-3.61 (D)	*Unknown*
		p.R1349Q	rs3745842	6, 8	0.3665	0.4433	0.5433	0.004 (B)	0.54 (T)	0.05 (N)	Common variant, unknown
		p.G1527H	rs3752246	1,2,3,4,5,7,8	0.12	0.25	0.321	0.004 (B)	0.54 (T)	0.1 (N)	*AD risk factor*
		p.Q1686R	rs4147918	3	0.1503	0.047	0.05	0.004 (B)	0.23 (T)	-0.44 (N)	*AD risk factor*
		p.A2045S	rs4147934	1,2,4,6,7,8	0.4220	0.7317	0.63	0.051 (B)	0.96 (T)	0.06 (N)	*Putative AD risk factor*
		p.F2071C	NA	3	0.0064	0.0025	0.003	0.5 (B)	0.46 (T)	-6.84 (D)	*Common mutation, unknown*
11	*CD33*	p.A14V	rs12459419	2, 5, 8	0.174437	0.2939	0.23	0.023 (B)	0.08 (T)	-2.12 (N)	*AD risk factor*
		p.R69G	rs2455069	6	0.058682	0.3577	0.34	0.009 (B)	0.33 (T)	-2.36 (N)	*Unknown*
		p.R98K	rs148118239	6	0.0001	0.004	0.0003	0.003 (B)	1 (T)	3.06 (N)	*unknown*
12	*PINK1*	p.Y253D	NA	6	NA	NA	NA	0.18 (B)	0.165 (T)	2.36 (N)	*unknown*
		p.A340P	rs3738136	1, 6	0.3054	0.09211	0.092	0.605 (D)	0.05 (D)	-3.91 (D)	May be risk factor for progressive supranuclear palsy
		p.N521T	rs1043424	1, 6	0.377010	0.09211	0.2811	0.005 (B)	0.24 (T)	-0.94 (N)	Unclear significance

13	*PARK2*	p.S167N	rs1801474	1, 3, 4, 5, 7	0.456592	0.06758	0.146	0.03 (B)	0.31 (T)	-1.45 (N)	*Benign variant*
		p.V380L	rs1801582	6, 8	0.044212	0.1646	0.145	0 (B)	0.37 (T)	-0.09 (N)	*Benign variant*
14	*LRRK2*	p.R50H	rs2256408	8/8	1.000000	0.9911	0.97	0 (B)	1 (T)	0.94 (N)	*Common variant, putative PD risk factor*
		p.N551K	rs7308720	4	0.152733	0.08607	0.102	0.972 (D)	0.005 (D)	-2.63 (D)	*Putative PD risk factor*
		p.R1398Q	rs7133914	8	0.136656	0.08412	0.0953	0.58 (D)	0.1 (T)	-1.19 (N)	*Putative PD risk factor/putative protective effect*
		p.R1628P	rs33949390	8	NA	NA	0.0005	0.899 (D)	0.04 (D)	-3.63 (D)	*PD risk factor*
		p.S1647T	rs11564148	1, 2, 3, 6,7, 8	0.260450	0.2983	0.2754	0.29 (B)	0.081 (T)	-0.15 (N)	*Possible PD risk factor*
		p.N2081D	rs33995883	2	NA	0.0009	0.0008	0 (B)	0.95 (T)	-0.15 (N)	*Common variant, may not be significant*
		p.M2397T	rs3761863	1, 2, 3, 4, 6,7, 8	0.418810	0.6240	0.548	0 (B)	0.466 (T)	-0.53 (N)	Possible PD risk factor
15	*SIGMAR1*	p.Q2P	rs1800866	1,3,5,8	0.319936	0.1840	0.195	0 (B)	0.343 (T)	0.03 (N)	*Possible risk factor*
16	*MAPT*	p.P513A	NA	2	NA	NA	NA	0.4 (B)	0.053 (T)	-0.53 (N)	*Unknown*
17	*ALS2*	p.V368M	NA	8/8	1.000000	0.9106	0.89	0.009 (B)	0.191 (T)	-0.06 (N)	*Common mutation, unknown*
18	*FIG4*	p.M364L	rs2295837	2	0.221061	0.0742	0.098	0.001 (B)	1 (T)	1.11 (N)	*Benign mutation*

		p.V654A	rs9885672	2,3,5,6,8	0.354502	0.2753	0.069	0 (B)	0.3 (T)	0.95 (N)	*Benign mutation*
19	*OPTN*	p.M98K	rs11258194	3,7,8	0.080	0.0451	0.0678	0.04 (B)	0.92 (T)	-0.06 (N)	*Involved in glaucoma*
		p.E316K	NA	8/8	NA	NA	NA	0.127 (B)	0.025 (D)	-1.06 (N)	*Common variant, may not be significant*
20	*SPG11*	p.N278S	NA	7	0.07479	0.00779	0.003	0.01 (B)	0.45 (T)	-0.85 (N)	*unknown*
		p.F463S	rs3759871	1,2,3,6,8	0.4791	0.4657	0.496	0.009 (B)	0.341 (B)	-1.27 (N)	Unknown
		p.I1250T	NA	1	NA	NA	NA	0.001 (B)	0.06 (T)	1.2 (N)	*unknown*
21	*CSF1R*	p.P54Q	NA	4	0.2901	0.00009	0.00008	0.15 (B)	0.06 (T)	-3.36 (D)	*Novel mutation, may be risk factor for AD*
		p.H362R	s10079250	1,2,3,4,7,8	0.29019	0.00009	0.067	0.15 (B)	0.06 (T)	-3.36 (D)	*Common mutation, possible cancer risk factor*
		p.L536V	rs55942044	6	NA	NA	NA	0.101 (B)	1.00 (T)	4.27 (N)	*Probable novel variant, predicted to be damaging*
22	*NOTCH3*	p.R1175W	rs200504060	8	0.012058	0.001047	0.0024	0.91 (D)	0.004 (D)	-5.05 (D)	*unknown*
		p.A2223V	rs1044009	8/8	0.590032	0.7591	0.6546	0.003 (B)	0.14 (T)	-1.46 (N)	*Common variant, unknown significance*
23	*PRNP*	p.M129V	rs1799990	7	0.024920	0.3078	0.26373	0.012 (B)	0.024 (D)	-0.66 (N)	*Pathogenic nature complicated*
		p. E219K	rs1800014	6	0.057074	0.008728	0.009	0.003 (B)	0.035 (D)	0.00 (N)	*Pathogenic nature complicated*
